# Inverse Potts model improves accuracy of phylogenetic profiling

**DOI:** 10.1093/bioinformatics/btac034

**Published:** 2022-01-21

**Authors:** Tsukasa Fukunaga, Wataru Iwasaki

**Affiliations:** Waseda Institute for Advanced Study, Waseda University, Tokyo 1690051, Japan; Department of Computer Science, Graduate School of Information Science and Technology, The University of Tokyo, Tokyo 1130032, Japan; Department of Integrated Biosciences, Graduate School of Frontier Sciences, The University of Tokyo, Chiba 2770882, Japan; Department of Biological Sciences, Graduate School of Science, The University of Tokyo, Tokyo 1130032, Japan; Department of Computational Biology and Medical Sciences, Graduate School of Frontier Sciences, The University of Tokyo, Chiba 2770882, Japan; Atmosphere and Ocean Research Institute, The University of Tokyo, Chiba 2770882, Japan; Institute for Quantitative Biosciences, The University of Tokyo, Tokyo 1130032, Japan; Collaborative Research Institute for Innovative Microbiology, The University of Tokyo, Tokyo 1130032, Japan

## Abstract

**Motivation:**

Phylogenetic profiling is a powerful computational method for revealing the functions of function-unknown genes. Although conventional similarity metrics in phylogenetic profiling achieved high prediction accuracy, they have two estimation biases: an evolutionary bias and a spurious correlation bias. While previous studies reduced the evolutionary bias by considering a phylogenetic tree, few studies have analyzed the spurious correlation bias.

**Results:**

To reduce the spurious correlation bias, we developed metrics based on the inverse Potts model (IPM) for phylogenetic profiling. We also developed a metric based on both the IPM and a phylogenetic tree. In an empirical dataset analysis, we demonstrated that these IPM-based metrics improved the prediction performance of phylogenetic profiling. In addition, we found that the integration of several metrics, including the IPM-based metrics, had superior performance to a single metric.

**Availability and implementation:**

The source code is freely available at https://github.com/fukunagatsu/Ipm.

**Supplementary information:**

Supplementary data are available at *Bioinformatics* online.

## 1 Introduction

Genome sequences of many species have been determined, and accordingly, many function-unknown genes have been discovered. Revealing the functions of these function-unknown genes is an important research topic, but it is too time-consuming to experimentally verify the functions of all the genes. Therefore, the computational predictions of these gene functions are essential, and various methods have long been developed in bioinformatics. Phylogenetic profiling is one such analysis method. In this method, when two ortholog groups (OGs) have similar occurrence patterns among species in a table of OGs, the two OGs are presumed to be functionally related (Kensche *et al.*, 2008; Moi *et al.*, 2020; Niu *et al.*, 2017; Pellegrini *et al.*, 1999; Stupp *et al.*, 2021; Tremblay *et al.*, 2021; Tsaban *et al.*, 2021). Although phylogenetic profiling was first proposed to detect protein–protein interactions, this method in principle captures any functional relationships between genes. Phylogenetic profiling has been widely used to estimate the functions of function-unknown genes in various phylogenetic groups from prokaryotes to eukaryotes (Kumagai *et al.*, 2018; Sherill-Rofe *et al.*, 2019).

In conventional phylogenetic profiling, similarities in occurrence patterns between two OGs are directly calculated from a table of OGs. This direct calculation implicitly assumes that the species included in the table of OGs are independent of each other. This assumption is, however, incorrect because the species have evolutionary relationships. In other words, the conventional calculation of similarity introduces an evolutionary bias in the estimation. Therefore, methods that consider a phylogenetic tree were proposed and showed good performance (Barker *et al.*, 2007; Cohen *et al.*, 2012; Moi *et al.*, 2020; Ta *et al.*, 2011; Vert, 2002).

Another possible estimation bias is the spurious correlation bias between two OGs. In statistics, spurious correlation means that two unrelated (or weakly related) variables appear to be strongly related due to the influence of confounding factors. As a simple example, suppose there are functional relationships between OGs A and B and OGs A and C, but no (or weak) functional relationship between OGs B and C. In this case, OGs B and C can show similar occurrence patterns by bypassing OG A, which is a confounding factor. In real cases, transitive correlations among many genes and evolutionary relationships between species result in complex patterns of spurious correlations. Ignoring the possibility of spurious correlations should negatively influence the accuracy of the function predictions, but few studies have analyzed the spurious correlation bias. Kim and Price considered the spurious correlation bias in phylogenetic profiling and showed that the bias could be reduced using partial correlation based on a Gaussian graphical model (Kim and Price, 2011). However, they did not explicitly deal with the evolutionary bias and implicitly assumed that tables of OGs follow the Gaussian distribution, but this assumption does not hold true for categorical data.

Metrics commonly used for phylogenetic profiling are mutual information (MI), correlation coefficients and Jaccard coefficients. These metrics are local metrics calculated from only two OG profiles, and the locality causes spurious correlations whose confounding factors are the other OGs. Therefore, we can reduce spurious correlation biases by using global metrics calculated from all OG profiles. The inverse Potts model (IPM), also called direct coupling analysis or evolutionary coupling (Cocco *et al.*, 2018), is an analysis method for categorical datasets to calculate global metrics. The IPM has been applied to various biological data analyses, such as protein–protein interaction prediction (Cong *et al.*, 2019; Weigt *et al.*, 2009), protein structure prediction (Marks *et al.*, 2011; Muscat *et al.*, 2020), neural data analysis (Schneidman *et al.*, 2006; Watanabe *et al.*, 2013) and genome-wide association studies (Schubert *et al.*, 2019; Skwark *et al.*, 2017), and has improved prediction performance. Recently, Croce *et al.* (2019) identified physically interacting protein domain pairs by applying the IPM to tabular data whose rows and columns are species and protein domains. They revealed that the IPM could detect interacting domain pairs with higher accuracy than simple correlation coefficients. Their study was similar to phylogenetic profiling, but their goal was to predict domain-domain interactions and not to estimate gene functional associations.

In this study, we applied the IPM to phylogenetic profiling to accurately predict gene functions. We used direct information (DI) calculated based on the IPM as the global metric. We also developed DI that considers phylogenetic tree information to explicitly deal with the evolutionary bias. We investigated the performance of several metrics in phylogenetic profiling, and verified that the IPM-based metrics improved the accuracy of predicting gene functions. In addition, we found that the integration of several metrics, including the IPM-based metrics, has superior performance to a single metric.

## 2 Materials and methods

### 2.1 Input data

Two settings were assumed in our study: standard and evolutionary settings. Under the standard setting, the input data for our method is a table of OGs *D*, which consists of *N* species and *L* OGs. $D_{i,j}$ represents whether species *i* has OG *j* and takes either 0 or 1. Under the evolutionary setting, the input data for our method is a table of OG gain/losses *D*, which consists of *N* phylogenetic tree branches and *L* OGs. Given a phylogenetic tree and a table of OGs, gene-content evolutionary history is reconstructed to infer gene gain/losses on each branch of the tree. $D_{i,j}$ represents whether the gain/loss events of OG *j* occurred at edge *i*. The value takes 0, 1 or 2, indicating that there are no gene gain/loss events, gene gain events or gene loss events, respectively.

For the experiments in this study, we used three empirical datasets: archaea (domain), micrococcales (order) and fungi (kingdom) (Fukunaga and Iwasaki, 2021). The tables of OGs were prepared by preprocessing OG data in the STRING database (Szklarczyk *et al.*, 2019). We ignored gene copy number information and removed OGs that were shared by <10% or more than 90% of the species to reduce the computational time to prepare *D*. The proportions of remaining OGs were 24.7%, 20.0% and 16.8% in archaea, micrococcales and fungi datasets, respectively, because the dataset contained many OGs with few genes. The computational time of our method is proportional to the square of the number of OGs, thus this reduced the computational time by 95%. The removed OGs were expected not to have significant impacts on the results because of their low information content. The archaea, micrococcales and fungi datasets consisted of 151 species and 2875 OGs, 111 species and 1905 OGs, and 123 species and 5786 OGs, respectively. Under the evolutionary setting, we prepared *D* by reconstructing the gene-content evolutionary history for the three empirical datasets. We used Mirage (Fukunaga and Iwasaki, 2021) with the BDARD model (Kim and Hao, 2014) and the PM model (default parameters were used for the others). Phylogenetic trees were supplied by the Genome Taxonomy Database release 89 (Parks *et al.*, 2018) for the archaea and micrococcales datasets and the SILVA database release 111 (Yarza *et al.*, 2017; Yilmaz *et al.*, 2014) for the fungi dataset.

### 2.2 The IPM

We introduce ${\text{MI}}_{ab}$, which is the MI between OG *a* and OG *b*. The formula is as follows:

$${\text{MI}}_{ab}=\sum_{i=0}^{Q}\sum_{j=0}^{Q}f_{ab}(i,j)\ln\frac{f_{ab}(i,j)}{f_{a}(i)f_{b}(j)},$$

where $f_{a}(i)$ and $f_{ab}(i,j)$ are the relative frequencies of OG *a* taking *i* and OG *a* and OG *b* taking *i* and *j*, respectively, in the dataset *D*. *Q* is the maximum value that an OG can take (i.e. *Q *=* *1 under the standard setting and *Q *=* *2 under the evolutionary setting). The more OGs A and B depend on each other, the larger the ${\text{MI}}_{ab}$. If ${\text{MI}}_{ab}$ becomes 0, OGs A and B are completely independent. Note that ${\text{MI}}_{ab}$ can detect not only gene pairs with similar occurrence patterns but also those with anti-correlated relationships (i.e. if a genome contains one of the genes, it unlikely contains the other). Several previous studies showed that anti-correlation relationships also provide clues to functions of function-unknown genes (Croce *et al.*, 2019; Kim and Price, 2011; Morett *et al.*, 2003). We defined standard MI (SMI) and EMI as MI calculated under the standard and evolutionary settings, respectively.

${\text{MI}}_{ab}$ is a local metric calculated from only two OG profiles and is vulnerable to spurious correlations. Therefore, we calculated a global metric using all OG profiles based on the IPM. We first formulate the joint probabilities of all OGs as follows (Cocco *et al.*, 2018):

$$\begin{array}{c} P(x_{1},\ldots ,x_{L})\\ =\frac{1}{Z}\text{exp}\;\{\sum_{a=1}^{L}h_{a}(x_{a})+\sum_{a<b}J_{ab}(x_{a},x_{b})\},\\ Z=\sum_{\Omega }\;\text{exp}\;\{\sum_{a=1}^{L}h_{a}(x_{a})+\sum_{a<b}J_{ab}(x_{a},x_{b})\}. \end{array}$$

$P(x_{1},\ldots ,x_{L})$ is the joint probability that OG *a* takes *x_a_* for any *a*. $h_{a}(x_{a})$ is a weight parameter when OG *a* is *x_a_*, and $J_{ab}(x_{a},x_{b})$ is also a weight parameter when OG *a* is *x_a_* and OG *b* is *x_b_*. Ω is the set of all possible combinations that all OGs can take, and *Z* is a normalizing constant, which is called the partition function. This probabilistic model is obtained by deriving a model that maximizes entropy under the following constraints: $f_{a}(i)=p_{a}(i)$ for all *a* and *i* and $f_{ab}(i,j)=p_{ab}(i,j)$ for all *a*, *b*, *i* and *j*. $p_{a}(i)$ and $p_{ab}(i,j)$ are the marginal probabilities of $P(x_{1},\ldots ,x_{L})$ and represent the probabilities of OG *a* taking *i* and OG *a* and OG *b* taking *i* and *j*, respectively. This model is generally called the Potts model in statistical physics (when *Q *=* *1, this model is specifically called the Ising model). Note that this model is also a particular form of the Boltzmann machine or Markov random field.

In the derivation of the Potts model, the number of substantial constraints is $LQ+\frac{L(L-1)}{2}Q^{2}$ because $\sum_{i}f_{a}(i)=1$ and $\sum_{ij}f_{ab}(i,j)=1$ must be satisfied. On the other hand, the number of parameters in the model is $L(Q+1)+\frac{L(L-1)}{2}{\left(Q+1\right)}^{2}$, which is larger than the number of substantial constraints. This over-parameterization leads to the non-identification of the model. Therefore, it is necessary to introduce additional constraints on the parameters to reduce the degrees of freedom of the model. In this study, we used the following constraints, called lattice gas gauges, for ease of implementation (Cocco *et al.*, 2018):

$$h_{a}(0)=J_{ab}(0,i)=J_{ab}(i,0)=0\;\text{for all}\;a,b,i.$$

To calculate the parameters $h_{a}(i)$ and $J_{ab}(i,j)$ analytically, we need to count all the combinations in Ω. However, its computational cost can become too large when *L* is large because the number of combinations becomes large. Therefore, these parameters are learned from the dataset in an unsupervised manner (Section 2.3). Then, using the estimated parameters, the dependence between OG *a* and OG *b* is measured as ${\text{DI}}_{ab}$ as follows (Weigt *et al.*, 2009):

$$\begin{array}{c} {\text{DI}}_{ab}=\sum_{i=0}^{Q}\sum_{j=0}^{Q}P_{ab}^{\mathrm{dir}}(i,j)\ln\frac{P_{ab}^{\mathrm{dir}}(i,j)}{P_{a|b}^{\mathrm{dir}}(i)P_{b|a}^{\mathrm{dir}}(j)},\\ P_{ab}^{\mathrm{dir}}(i,j)=\frac{1}{Z_{ab}}\text{exp}\;\{h_{a}(i)+h_{b}(j)+J_{ab}(i,j)\},\\ Z_{ab}=\sum_{i,j}\;\text{exp}\;\{h_{a}(i)+h_{b}(j)+J_{ab}(i,j)\},\\ P_{a|b}^{\mathrm{dir}}(i)=\sum_{j}P_{ab}^{\mathrm{dir}}(i,j). \end{array}$$

This definition is slightly different from the original definition (Weigt *et al.*, 2009). In the original DI calculation, $f_{a}(i)$ was used instead of $P_{a|b}^{\mathrm{dir}}(i)$, and $h_{a}(i)$ was re-calculated from $f_{a}(i)=\sum_{b}P_{ab}^{\mathrm{dir}}(i,j)$. Similar to ${\text{MI}}_{ab}$, the more OGs A and B depend on each other, the larger ${\text{DI}}_{ab}$. Note that ${\text{DI}}_{ab}$ can also detect anti-correlated relationships. We defined standard DI (SDI) and EDI as the DI calculated under the standard and evolutionary settings, respectively.

In addition to DI, Frobenius norm (FN) and average product correction (APC) are widely used metrics to quantify dependencies between two elements in the IPM. These metrics are gauge-dependent quantities, and the best gauge is the zero-sum gauge (Ekeberg *et al.*, 2013). On the other hand, DI has gauge-independent characteristics (Ekeberg *et al.*, 2013). Because we used lattice-gas gauges in this study, we used DI instead of FN and APC for the metrics.

### 2.3 Parameter estimation method

To date, various algorithms have been developed to estimate the parameters of the Potts model, for example, mean-field approximation (Morcos *et al.*, 2011), pseudo-likelihood maximization (Ekeberg *et al.*, 2013), adaptive cluster expansion (Barton *et al.*, 2016) and Markov chain Monte Carlo (MCMC) methods (Figliuzzi *et al.*, 2018). There is an approximate trade-off between the computational speed and estimation accuracy in these methods, that is, more accurate methods require longer run times. In this study, we focused on the estimation accuracy, and used the persistent contrastive divergence (PCD) method (Hinton, 2002; Tieleman, 2008), which is a variant of the MCMC method. We maximized the likelihood with the L2-regularization term to avoid overfitting the data in the PCD method.

The algorithm for the PCD method is as follows. We first randomly sample *K* samples with replacement from the dataset *D*, and let the initial sampled dataset be *D*^0^. In this study, we set *K* to 200. In addition, we set all the initial parameters to 0. Next, we obtained the dataset *D*^1^ from *D*^0^ and the initial parameters based on the following Gibbs sampler:

$$D_{i,j}^{1}∼P(x_{j}|D_{i,1}^{1},\ldots ,D_{i,j-1}^{1},D_{i,j+1}^{0},\ldots ,D_{i,L}^{0}).$$

This sampling was performed *LK* times to obtain *D*^1^. Then, we calculated ${\hat{f}}_{a}(i)$ and ${\hat{f}}_{ab}(i,j)$, which are the relative frequencies of OG *a* taking *i* and OG *a* and OG *b* taking *i* and *j* in the dataset *D*^1^, respectively. Subsequently, the model parameters were updated using the following formula:

$$\begin{array}{c} h_{a}(i)\leftarrow h_{a}(i)+\epsilon (f_{a}(i)-{\hat{f}}_{a}(i))-2\lambda h_{a}(i)\\ J_{ab}(i,j)\leftarrow J_{ab}(i,j)+\epsilon (f_{ab}(i,j)-{\hat{f}}_{ab}(i,j))-2\lambda J_{ab}(i,j). \end{array}$$

$2\lambda h_{a}(i)$ and $2\lambda J_{ab}(i,j)$ are the L2-regularization terms, and we used any 0, 0.01, 0.05, 0.1, 0.5, 1.0 or 5.0 as *λ*. Note that *λ *= 0 indicates simple likelihood maximization without the regularization terms. *ϵ* represents a learning rate, and we set either 0.01 or 0.001 as *ϵ*. After parameter estimation, we sampled dataset *D*^2^ from *D*^1^ using the estimated parameters. We finally adopted parameters after repeating the Gibbs sampling and the parameter update 3000 times.

### 2.4 Evaluation method

We assessed the prediction performance of each metric using association scores between two OGs provided in the STRING database (Szklarczyk *et al.*, 2019). The association scores in the STRING database were calculated by considering gene neighborhood conservation, gene fusion, co-expression, protein interaction experiments, other databases, text mining and occurrence patterns. Because occurrence patterns should not be used in the assessment, we recalculated the association scores by ignoring the occurrence pattern similarities. If the recalculated association score of an OG pair was larger than the threshold *th*, we regarded the OG pair as positive data; otherwise, we regarded it as negative data. We used the threshold *th* from 0.5 to 0.9 in 0.1 increments. The sizes of each dataset are listed in Supplementary Table S1. Note that the association scores of 0.7 and 0.9 are the lower limits of high and highest confidences, respectively, in the STRING database.

We first investigated the overall discrimination performance of each metric using the area under the receiver operating characteristic curve (AUC) scores. The AUC scores were calculated using the pROC R package (Robin *et al.*, 2011). We also assessed the prediction accuracy of the OG pairs that were highly ranked by each metric. Specifically, we defined the highly ranked OG pairs as the top *M* OG pairs in each metric, and calculated the positive predictive values (PPVs) of these pairs (at *th *=* *0.7). We used 100, 500, 1000, 5000 or 10 000 as *M*. In addition, we evaluated AUPR scores using the PRROC package for the analysis of highly ranked OG pairs (Grau *et al.*, 2015).

## 3 Results

### 3.1 Performances of single metrics

We first assessed the overall discrimination performance of the four metrics (SMI, EMI, SDI and EDI) based on the AUC scores. We investigated 14 combinations of seven *λ* values and two *ϵ* values as IPM hyperparameters for calculating the SDI and EDI. In the following analyses, we used the hyperparameters showing the best AUC score for each dataset and each *th* value. The AUC scores are listed in Supplementary Tables S2–S7. Both hyperparameters had a large impact on the prediction performance. In addition, the optimal hyperparameters differed depending on the dataset and the *th* value. We also found that the optimal hyperparameter *λ* was not 0.0 in many cases. This result means that L2-regularization was effective for achieving high discrimination performance.

We checked the distribution of each metric after normalizing the maximum value to 1.0, and calculated the skewness (Supplementary Figs S1 and S2). We found that the distribution was skewed to the right in all cases, that is, only a portion of OG pairs obtained high scores in each metric. In addition, we discovered that the consideration of both gene-content evolutionary history and usage of the IPM increases the skewness of the distribution. These results suggest that the biases in SMI were reduced by the reconstruction of the gene content history and the IPM method.

**Fig. 1.**
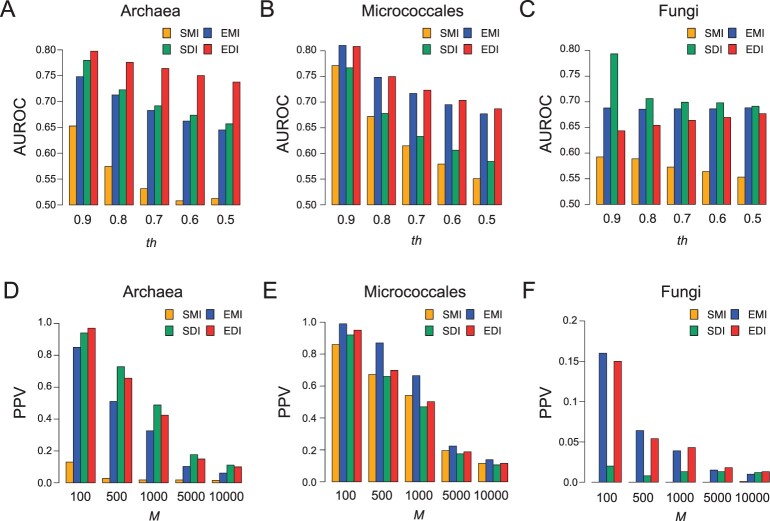
(**A**–**C**) Overall discrimination performances of each metric using the AUC scores. The *x*-axis represents the *th* value, which defines positive dataset. The *y*-axis represents the AUROC score. (A), (B) and (C) panels represent results for the archaea, micrococcales and fungi datasets, respectively. (**D**–**F**) Prediction performances for highly ranked OG pairs of each metric (*th *=* *0.7). The *x*-axis represents the *M* value. The *y*-axis represents the PPV. (D), (E) and (F) panels represent results for the archaea, micrococcales and fungi datasets, respectively. The yellow, blue, green and red colors represent SMI, EMI, SDI and EDI, respectively

Figure 1A–C shows the results of the AUC analyses. We found that EMI outperformed SMI in all cases, which suggests that gene content history reconstruction is highly effective in phylogenetic profiling, which is consistent with previous studies (Barker *et al.*, 2007; Moi *et al.*, 2020; Ta *et al.*, 2011). SDI was always better than SMI, except for one case where similar performances were obtained (*th *=* *0.9 in the micrococcales dataset). These results also suggest that the IPM is valuable for reducing biases containing spurious correlation and evolutionary biases. EDI showed the best performance in the archaea and micrococcales datasets, except for the same case where EMI and EDI showed comparable performances. On the other hand, SDI showed the best performance in the fungi dataset. A cause of the worse performance of EDI in the fungi dataset may be insufficient gene annotation. Although the recalculated STRING scores used gene neighborhood conservation and gene fusion, they are not effective in estimating eukaryotic protein functional relationships. We found that the proportion of positive data was much lower for the fungi dataset than for the other datasets (Supplementary Table S1). This suggests that many functionally related OG pairs were not annotated with high association scores in the fungi dataset.

We next investigated the prediction accuracies of highly ranked (top *M*) OG pairs for each metric (Fig. 1D–F). In almost all cases, SMI exhibited the worst or near-worst performance. On the other hand, the best-performing metrics depended on the datasets and *M*. For example, when *M* was 1000, SDI, EMI and EDI showed the highest PPVs for the archaea, micrococcales and fungi datasets, respectively. We confirmed that AUPR scores, where the top-scored prediction has large effects, showed the similar tendency with the PPV scores (Supplementary Fig. S3). Thus, the reconstruction of gene content history and the IPM method generally increase performances, although whether EMI, SDI or EDI performs the best depends on the case.

### 3.2 Performances of integrated metrics

Because highly ranked OG pairs estimated by EMI, SDI and EDI showed the best performance depending on the conditions, we next investigated whether their integration showed better performance. There are four combination types for the integration: EMI and SDI, EMI and EDI, SDI and EDI, and all three metrics. For the integration, we first ordered the OG pairs in descending order by their scores for EMI, SDI and EDI. Then, for each combination, we sorted the OG pairs by any of the integration types that are the maximum, average or minimum values of their ranks in all metrics under consideration.

**Fig. 2.**
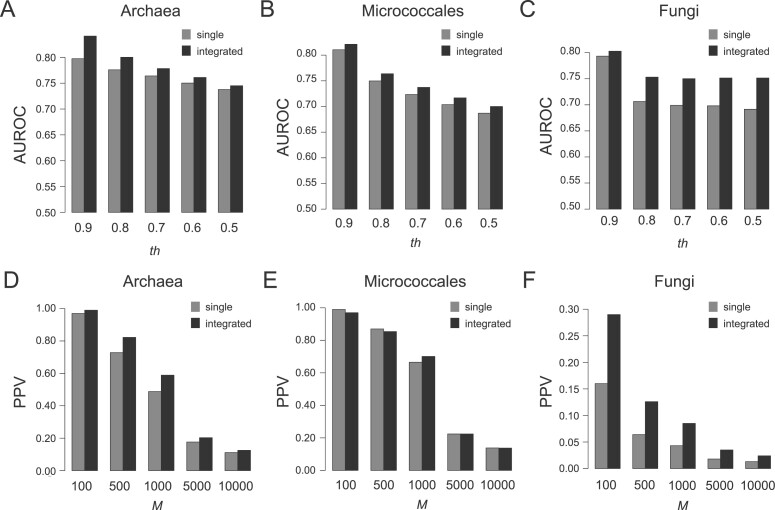
(**A**–**C**) Overall discrimination performances of integrated metrics using the AUC scores. The *x*-axis represents the *th* value, which defines positive dataset. The *y*-axis represents the AUROC score. (A), (B) and (C) panels represent results for the archaea, micrococcales and fungi datasets, respectively. (**D**–**F**) Prediction performances for highly ranked OG pairs of integrated metrics (*th *=* *0.7). The *x*-axis represents the *M* value. The *y*-axis represents the PPV. (D), (E) and (F) panels represent results for the archaea, micrococcales and fungi datasets, respectively. The gray and black colors represent the highest single metric and integrated metric, respectively

We investigated the AUC, PPV and AUPR performances of 12 integrated metrics comprising four combination types and three integration types (Supplementary Tables S8–S16). We found that the best condition for the integrated metrics depends on the dataset and the threshold (*th* or *M*). As a general trend, while the integration by the minimum values showed the highest scores in the AUC analyses, the integration by the average values achieved the highest scores in the PPV and AUPR analyses. In addition, we found that the highest integrated metrics performed better than the highest single metrics in many cases (Fig. 2 and Supplementary Fig. S4). These results strongly suggest that while EMI, SDI and EDI are good metrics, they also lose useful information in functional estimation in its own way, which could be salvaged by their integration.

### 3.3 Examples of the detected OG pairs

Finally, as examples of the highly ranked OG pairs, we show lists of the top five ranked OG pairs by the integration of all three metrics (Table 1). We used the average value as the integration type and regarded the value as the prediction score. Except for two cases, these OG pairs had recalculated STRING association scores above 0.9, which means that functional associations had the highest confidence. Most of these gene pairs had known functional relationships. For example, the first rank in the archaea dataset was a pair of *ZnuA* and *ZnuB*, which are components of the ABC-type zinc uptake system. As another example, the fifth rank in the micrococcales dataset was a pair of *DnaC*, which is involved in DNA replication, and COG4584, a transposase.

**Table 1.** The lists of the top five OG pairs detected by the combination of all three metrics

Taxonomy	Rank	OG1	OG2	Prediction score	STRING score
	1	COG0803 (*ZnuA*)	COG1108 (*ZnuB*)	10.0	0.992
	2	COG1203 (*Cas3*)	COG1688 (*Cas5*)	14.7	0.996
Archaea	3	COG1108 (*ZnuB*)	COG1121 (*ZnuC*)	17.3	0.994
	4	COG2998 (*TupA*)	COG4662 (*TupA)*	21.3	0.999
	5	COG1336 (*Cmr4*)	COG1604 (*Cmr6*)	24.0	0.999
	1	COG3181 (*TctC*)	COG3333 (*TctA*)	1.7	0.989
	2	COG1135 (*AbcC*)	COG2011 (*MetP*)	7.0	0.995
Micrococcales	3	COG1464 (*NlpA*)	COG2011 COG2011 (*MetP*)	10.7	0.996
	4	COG1135 (*AbcC*)	COG1464 (*NlpA*)	12.3	0.987
	5	COG1484 (*DnaC*)	COG4584	12.7	0.986
	1	COG0043 (*UbiD*)	COG0163 (*UbiX*)	34.3	0.998
	2	KOG4501	NOG13474	143.3	0.0
Fungi	3	COG5441	COG5564	620.3	0.988
	4	COG0843 (*CyoB*)	COG1290 (*QcrB*)	682.3	0.969
	5	COG2051 (*RPS27A*)	KOG3504	774.7	0.0

The first exceptional pair with the recalculated STRING score of 0.0 was KOG4501 and NOG13474, which was ranked second in the fungi dataset. We further investigated the relationship between these two genes and found that they showed an anti-correlated relationship. An anti-correlated relationship is also a clue for gene-function estimation as explained earlier, and it should be noted that the recalculated STRING scores based on gene neighborhood conservation, gene fusion, co-expression, protein-interaction experiments, other databases, and text mining cannot detect signals of anti-correlated relationships. While the human gene belonging to KOG4501 has a known function that is involved in DNA damage repair (Brickner *et al.*, 2017), NOG13474 is a function-unknown gene. We argue that NOG13474 may have a DNA damage repair function as a complement of KOG4501. In addition, the second exceptional pair was COG2051 and KOG3504, which was ranked fifth in the fungi dataset. Because both these OGs are ribosomal proteins, the recalculated STRING score may suggest the insufficient annotation.

## 4 Discussion

In this study, we evaluated the effectiveness of IPM in the phylogenetic profiling analysis. We constructed four metrics, SMI, EMI, SDI and EDI, based on whether a phylogenetic tree and the IPM were used. We then investigated the performance of the four metrics using the STRING datasets. We showed that SDI and EDI had the best performances in many cases. In addition, we revealed that predictions based on the combinations of EMI, SDI and EDI showed higher performance than predictions based on a single metric. These results demonstrated that the IPM is a powerful approach in phylogenetic profiling.

Although even simple combinations of the metrics yielded good prediction results, more sophisticated methods of combining the metrics may provide better prediction results, for example, machine learning methods. A similar concept was proposed in studies on protein structure prediction based on IPM (Jones *et al.*, 2015; Wang *et al.*, 2017). These studies integrated various scores, such as co-evolutionary information using IPM, and predicted solvent accessibility information using supervised machine learning methods, such as deep learning.

Theoretically, phylogenetic profiling methods detect any functional relationships regardless of whether they are physical or functional interactions. Thus, to discriminate types of identified relationships, other bioinformatic approaches need to be additionally employed. For example, by taking advantage of the recent breakthroughs of the AlphaFold2 (Jumper *et al.*, 2021) and AlphaFold-Multimer tools (Evans *et al.*, 2021), phylogenetic profiling will be used to specifically identify physically interacting protein pairs. We envision combining our method with the accurate protein structure prediction methods in the near future.

We assumed that the input phylogenetic tree and gene content evolutionary history were correct when calculating EMI and EDI. However, they were estimations and intrinsically subject to uncertainty. Such uncertainty should decrease the accuracy of phylogenetic profiling analysis in general (Hamada, 2014). One solution is to consider the distribution of the estimates by calculating the expected values (instead of counts) of gene gains and losses for each phylogenetic branch. Cohen *et al.* (2012, 2013) adopted this approach, but a comparison with other methods has not been conducted and further studies are required. Because this extension requires the use of continuous data, the Gaussian graphical model will need to be used for considering spurious correlations, instead of the Potts model for categorical data (Stein *et al.*, 2015).

In this study, we analyzed only the relationships between two OGs; however, many OGs have higher-order functional relationships among three or more OGs (such as multi-protein complexes). Several studies have focused on the logic relationships of three OGs in phylogenetic profiling (Bowers *et al.*, 2004; Fukunaga and Iwasaki, 2020; Zhang *et al.*, 2006). An example of a logic relationship is C=A∧B for OGs A, B and C, which means that OG C needs both OGs A and B for its function. To date, logic relationship analysis in phylogenetic profiling used local metrics, thus the detection of such higher-order functional relationships based on global metrics is an essential future task. Technically, it is not difficult to extend the Potts model to include (more than) ternary relationships (Schmidt and Hamacher, 2017), but efficient parameter estimation and construction of large-scale datasets for precise parameter estimation will be difficult.

## Supplementary Material

btac034_Supplementary_Materials
