# Supplementary material for: Inverse Potts model improves accuracy of phylogenetic profiling
Source: Bioinformatics. 2022 Jan 21;38(7):1794–800. doi: 10.1093/bioinformatics/btac034 (PMC8963296; doi:10.1093/bioinformatics/btac034)
Supplement: btac034_Supplementary_Materials [file btac034_supplementary_materials.pdf]

Supplementary Materials for Inverse Potts model improves accuracy of phylogenetic profiling

Tsukasa Fukunaga and Wataru Iwasaki

Supplementary Figures

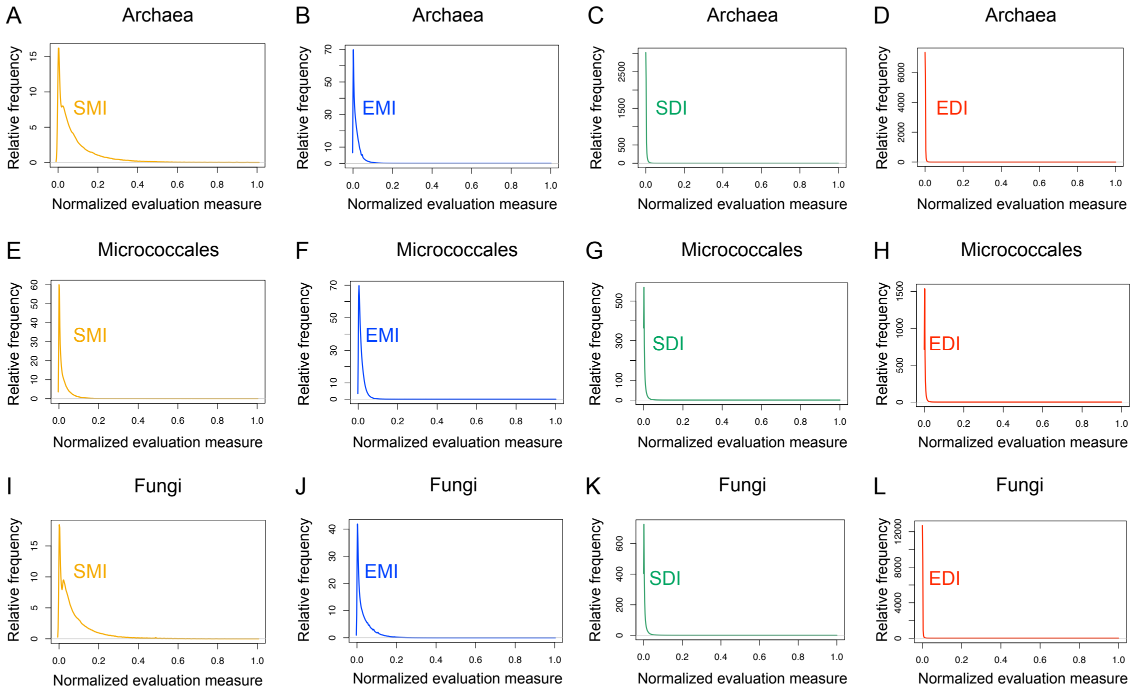

Fig. S1 The distributions of each evaluation metric. The x-axis and the y-axis represent the normalized evaluation metric and the relative frequency, respectively. The yellow, blue, green, and red colors represent the SMI, the EMI, the SDI, and the EDI, respectively. Distributions of (A) the SMI, (B) the EMI, (C) the SDI, and (D) the EDI for the archaea dataset are shown. In addition, distributions of (E) the SMI, (F) the EMI, (G) the SDI, and (H) the EDI for the micrococcales dataset are shown. Furthermore, distributions of (I) the SMI, (J) the EMI, (K) the SDI, and (L) the EDI for the fungi dataset are shown.

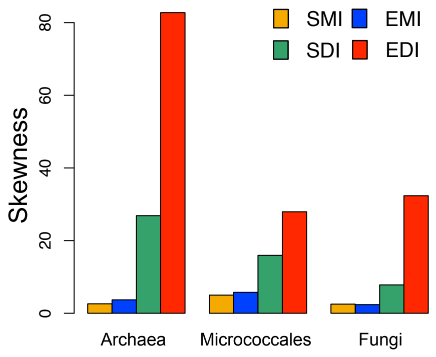

Fig. S2 The skewnesses of distributions of each evaluation metric. The yellow, blue, green, and red colors represent the SMI, the EMI, the SDI and the EDI, respectively. The y-axis represents the skewness.

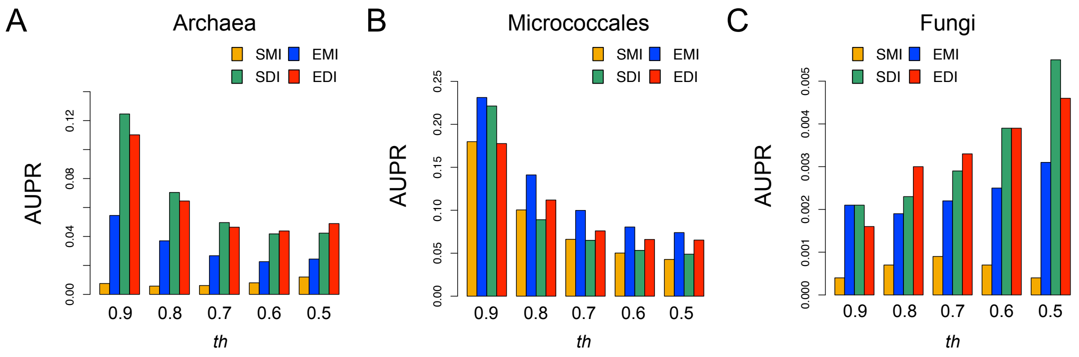

Fig. S3 Prediction performances of each evaluation metric using the AUPR scores. The x-axis represents the *th* value, which defines positive dataset. The y-axis represents the AUPR score. (A), (B), and (C) panels represent results for the archaea, micrococcales, and fungi datasets, respectively.

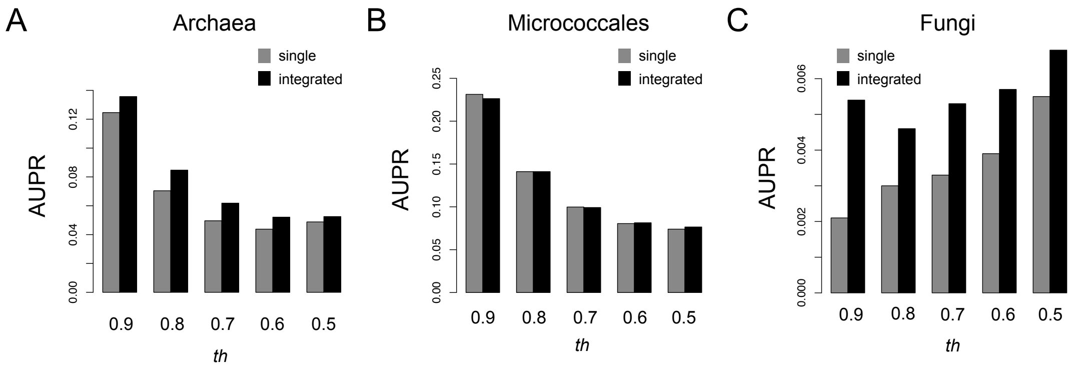

Fig. S4 Prediction performances of integrated evaluation metrics using the AUPR scores. The x-axis represents the *th* value, which defines positive dataset. The y-axis represents the AUPR score. (A), (B), and (C) panels represent results for the archaea, micrococcales, and fungi datasets, respectively.

Supplementary Tables

Table S1 The dataset size for each dataset

taxonomic group	threshold	positive data	negative data
Archaea	0.9	2912	4128463
	0.8	7548	4123827
	0.7	15112	4116263
	0.6	26416	4104959
	0.5	46717	4084658
Micrococcales	0.9	2426	1811134
	0.8	5567	1807993
	0.7	9953	1803607
	0.6	16666	1796894
	0.5	29135	1784425
Fungi	0.9	3101	16732904
	0.8	6415	16729590
	0.7	8968	16727037
	0.6	12668	16723337
	0.5	18871	16717134

Table S2 The AUC scores of SDI with varying parameters in the archaea data set

(λ, ϵ)	(0.0, 0.01)	(0.0, 0.001)	(0.01, 0.01)	(0.01, 0.001)	(0.05, 0.01)	(0.05, 0.001)	(0.1, 0.01)	(0.1, 0.001)
$th = 0.9$	0.7792	0.7785	0.7782	0.7784	0.7774	0.7787	0.7769	**0.7799**
$th = 0.8$	**0.7228**	0.7097	0.7225	0.7097	0.7204	0.7100	0.7169	0.7112
$th = 0.7$	**0.6918**	0.6708	0.6917	0.6708	0.6878	0.6709	0.6819	0.6718
$th = 0.6$	0.6735	0.6511	**0.6736**	0.6510	0.6687	0.6511	0.6627	0.6518
$th = 0.5$	**0.6568**	0.6327	**0.6568**	0.6326	0.6518	0.6324	0.6454	0.6333

(λ, ϵ)	(0.5, 0.01)	(0.5, 0.001)	(1.0, 0.01)	(1.0, 0.001)	(5.0, 0.01)	(5.0, 0.001)
$th = 0.9$	0.7796	0.7790	0.7797	0.7759	0.7489	0.7498
$th = 0.8$	0.7072	0.7061	0.6980	0.6957	0.6610	0.6609
$th = 0.7$	0.6667	0.6656	0.6575	0.6541	0.6255	0.6256
$th = 0.6$	0.6469	0.6459	0.6389	0.6352	0.6078	0.6075
$th = 0.5$	0.6280	0.6265	0.6189	0.6151	0.5889	0.5883

The bold values are the highest scores in each column.

Table S3 The AUC scores of SDI with varying parameters in the micrococcales data set.

(λ, ϵ)	(0.0, 0.01)	(0.0, 0.001)	(0.01, 0.01)	(0.01, 0.001)	(0.05, 0.01)	(0.05, 0.001)	(0.1, 0.01)	(0.1, 0.001)
$th = 0.9$	0.7529	0.7614	0.7550	0.7614	0.7613	0.7624	0.7630	0.7631
$th = 0.8$	0.6723	0.6734	0.6746	0.6734	**0.6779**	0.6739	0.6767	0.6740
$th = 0.7$	0.6278	0.6267	0.6306	0.6266	**0.6330**	0.6267	0.6308	0.6265
$th = 0.6$	0.6020	0.5969	0.6047	0.5969	**0.6063**	0.5966	0.6030	0.5962
$th = 0.5$	0.5809	0.5742	0.5837	0.5742	**0.5844**	0.5740	0.5807	0.5734

(λ, ϵ)	(0.5, 0.01)	(0.5, 0.001)	(1.0, 0.01)	(1.0, 0.001)	(5.0, 0.01)	(5.0, 0.001)
$th = 0.9$	**0.7669**	0.7668	0.7616	0.7619	0.7255	0.7266
$th = 0.8$	0.6734	0.6733	0.6643	0.6646	0.6309	0.6297
$th = 0.7$	0.6229	0.6226	0.6126	0.6123	0.5868	0.5861
$th = 0.6$	0.5900	0.5892	0.5788	0.5789	0.5607	0.5603
$th = 0.5$	0.5657	0.5650	0.5551	0.5553	0.5434	0.543

The bold values are the highest scores in each column.

Table S4 The AUC scores of SDI with varying parameters in the fungi data set.

(λ, ϵ)	(0.0, 0.01)	(0.0, 0.001)	(0.01, 0.01)	(0.01, 0.001)	(0.05, 0.01)	(0.05, 0.001)	(0.1, 0.01)	(0.1, 0.001)
$th = 0.9$	0.7308	0.7295	0.7523	0.7391	0.7434	0.7292	0.7397	0.7346
$th = 0.8$	0.6565	0.6498	0.6721	0.6579	0.6697	0.6515	0.6671	0.6563
$th = 0.7$	0.6518	0.6428	0.6667	0.6506	0.6651	0.6443	0.6630	0.6489
$th = 0.6$	0.6499	0.6406	0.6660	0.6477	0.6629	0.6416	0.6609	0.6457
$th = 0.5$	0.6483	0.6367	0.6631	0.6433	0.6601	0.6375	0.6582	0.6408

$(\lambda,\, \epsilon)$	(0.5, 0.01)	(0.5, 0.001)	(1.0, 0.01)	(1.0, 0.001)	(5.0, 0.01)	(5.0, 0.001)
$th = 0.9$	0.7697	0.7359	**0.7934**	0.7477	0.7245	0.7709
$th = 0.8$	0.6882	0.6586	**0.7064**	0.6643	0.6515	0.6701
$th = 0.7$	0.6783	0.6474	**0.6991**	0.6524	0.6462	0.6553
$th = 0.6$	0.6755	0.6427	**0.6981**	0.6481	0.6480	0.6535
$th = 0.5$	0.6687	0.6338	**0.6914**	0.6366	0.6472	0.6416

The bold values are the highest scores in each column.

Table S5 The AUC scores of EDI with varying parameters in the archaea data set.

$(\lambda,\, \epsilon)$	(0.0, 0.01)	(0.0, 0.001)	(0.01, 0.01)	(0.01, 0.001)	(0.05, 0.01)	(0.05, 0.001)	(0.1, 0.01)	(0.1, 0.001)
$th = 0.9$	0.7954	0.7573	**0.7974**	0.7566	0.7901	0.7570	0.7804	0.7560
$th = 0.8$	0.7751	0.7307	**0.7759**	0.7305	0.7669	0.7310	0.7555	0.7305
$th = 0.7$	**0.7640**	0.7118	0.7635	0.7119	0.7523	0.7122	0.7391	0.7117
$th = 0.6$	**0.7502**	0.6950	0.7493	0.6952	0.7374	0.6957	0.7234	0.6954
$th = 0.5$	**0.7378**	0.6819	0.7365	0.6819	0.7249	0.6824	0.7114	0.6822

$(\lambda,\, \epsilon)$	(0.5, 0.01)	(0.5, 0.001)	(1.0, 0.01)	(1.0, 0.001)	(5.0, 0.01)	(5.0, 0.001)
$th = 0.9$	0.7374	0.7341	0.6998	0.6993	0.5935	0.6005
$th = 0.8$	0.7140	0.7094	0.6732	0.6750	0.5606	0.5655
$th = 0.7$	0.6950	0.6915	0.6542	0.6555	0.5367	0.5398
$th = 0.6$	0.6781	0.6751	0.6363	0.6379	0.5171	0.5207
$th = 0.5$	0.6640	0.6611	0.6210	0.6228	0.5061	0.5083

The bold values are the highest scores in each column.

Table S6 The AUC scores of EDI with varying parameters in the micrococcales data set.

$(\lambda,\, \epsilon)$	(0.0, 0.01)	(0.0, 0.001)	(0.01, 0.01)	(0.01, 0.001)	(0.05, 0.01)	(0.05, 0.001)	(0.1, 0.01)	(0.1, 0.001)
$th = 0.9$	0.7941	0.8043	0.7969	0.8044	0.8037	0.8042	0.8085	**0.8036**
$th = 0.8$	0.7397	0.7434	0.7423	0.7438	0.7481	0.7438	0.7497	**0.7435**
$th = 0.7$	0.7160	0.7172	0.7187	0.7175	0.7233	**0.7179**	0.7229	0.7180
$th = 0.6$	0.6978	0.6981	0.7005	0.6983	0.7036	**0.6988**	0.7031	0.6990
$th = 0.5$	0.6818	0.6815	0.6847	0.6817	0.6869	**0.6825**	0.6859	0.6828

(λ, ϵ)	(0.5, 0.01)	(0.5, 0.001)	(1.0, 0.01)	(1.0, 0.001)	(5.0, 0.01)	(5.0, 0.001)
$th = 0.9$	0.7966	0.7964	0.7811	0.7806	0.7032	0.7119
$th = 0.8$	0.7358	0.7363	0.7201	0.7211	0.6393	0.6391
$th = 0.7$	0.7106	0.7116	0.6985	0.6995	0.6140	0.6167
$th = 0.6$	0.6935	0.6943	0.6828	0.6840	0.5993	0.6016
$th = 0.5$	0.6789	0.6793	0.6699	0.6711	0.5861	0.5886

The bold values are the highest scores in each column.

Table S7 The AUC scores of EDI with varying parameters in the fungi data set.

(λ, ϵ)	(0.0, 0.01)	(0.0, 0.001)	(0.01, 0.01)	(0.01, 0.001)	(0.05, 0.01)	(0.05, 0.001)	(0.1, 0.01)	(0.1, 0.001)
$th = 0.9$	0.6354	0.6194	**0.6435**	0.6116	0.6432	0.6233	0.6380	0.6253
$th = 0.8$	0.6459	0.6370	**0.6541**	0.6316	**0.6541**	0.6404	0.6512	0.6421
$th = 0.7$	0.6568	0.6445	**0.6640**	0.6394	0.6626	0.6479	0.6589	0.6495
$th = 0.6$	0.6628	0.6451	**0.6695**	0.6399	0.6671	0.6483	0.6617	0.6499
$th = 0.5$	0.6696	0.6509	**0.6769**	0.6462	0.6735	0.6540	0.6674	0.6554

(λ, ϵ)	(0.5, 0.01)	(0.5, 0.001)	(1.0, 0.01)	(1.0, 0.001)	(5.0, 0.01)	(5.0, 0.001)
$th = 0.9$	0.5946	0.5911	0.5513	0.5510	0.5341	0.5425
$th = 0.8$	0.6222	0.6203	0.5898	0.5916	0.4934	0.5081
$th = 0.7$	0.6310	0.6294	0.6008	0.6028	0.5169	0.5179
$th = 0.6$	0.6311	0.6291	0.6013	0.6031	0.5181	0.5181
$th = 0.5$	0.6379	0.6361	0.6097	0.6116	0.5246	0.5235

The bold values are the highest scores in each column.

Table S8 The AUC scores of integrated evaluation metrics in the archaea data set.

	EMI&SDI max	EMI&SDI avg	EMI&SDI min	EMI&EDI max	EMI&EDI avg	EMI&EDI min	SDI&EDI max	SDI&EDI avg
$th = 0.9$	0.7703	0.7993	0.8027	0.7804	0.7913	0.7858	0.7728	0.8162
$th = 0.8$	0.7297	0.7566	0.7540	0.7535	0.7662	0.7610	0.7405	0.7818
$th = 0.7$	0.7030	0.7264	0.7182	0.7351	0.7463	0.7382	0.7220	0.7630
$th = 0.6$	0.6864	0.7064	0.6938	0.7189	0.7289	0.7192	0.7073	0.7477
$th = 0.5$	0.6709	0.6833	0.6734	0.7048	0.7136	0.7026	0.6935	0.7334

	SDI&EDI min	all max	all avg	all min
$th = 0.9$	**0.8412**	0.7721	0.8149	0.8267
$th = 0.8$	**0.8002**	0.7382	0.7839	0.7873
$th = 0.7$	**0.7783**	0.7177	0.7623	0.7580
$th = 0.6$	**0.7611**	0.7026	0.7448	0.7364
$th = 0.5$	**0.7451**	0.6883	0.7288	0.7172

The bold values are the highest scores in each column.

Table S9 The AUC scores of integrated evaluation metrics in the micrococcales data set.

	EMI&SDI max	EMI&SDI avg	EMI&SDI min	EMI&EDI max	EMI&EDI avg	EMI&EDI min	SDI&EDI max	SDI&EDI avg
$th = 0.9$	0.7918	0.8070	0.8098	0.8040	0.8144	**0.8211**	0.7850	0.8026
$th = 0.8$	0.7180	0.7396	0.7438	0.7453	0.7574	**0.7637**	0.7146	0.7371
$th = 0.7$	0.6796	0.7027	0.7066	0.7161	0.7302	**0.7372**	0.6789	0.7039
$th = 0.6$	0.6554	0.6786	0.6817	0.6949	0.7096	**0.7167**	0.6551	0.6810
$th = 0.5$	0.6343	0.6583	0.6613	0.6768	0.6923	**0.6999**	0.6351	0.6614

	SDI&EDI min	all max	all avg	all min
$th = 0.9$	0.8065	0.7906	0.8161	0.8208
$th = 0.8$	0.7433	0.7211	0.7553	0.7591
$th = 0.7$	0.7106	0.6846	0.7241	0.7260
$th = 0.6$	0.6880	0.6608	0.7019	0.7028
$th = 0.5$	0.6686	0.6400	0.6830	0.6836

The bold values are the highest scores in each column.

Table S10 The AUC scores of integrated evaluation metrics in the fungi data set.

	EMI&SDI max	EMI&SDI avg	EMI&SDI min	EMI&EDI max	EMI&EDI avg	EMI&EDI min	SDI&EDI max	SDI&EDI avg
$th = 0.9$	0.7296	0.7822	0.7930	0.6514	0.6791	0.6922	0.7024	0.7636
$th = 0.8$	0.6927	0.7340	0.7445	0.6570	0.6833	0.6957	0.6669	0.7204
$th = 0.7$	0.6918	0.7312	0.7391	0.6649	0.6890	0.6992	0.6703	0.7221
$th = 0.6$	0.6924	0.7315	0.7381	0.6674	0.6921	0.7028	0.6727	0.7247
$th = 0.5$	0.6917	0.7300	0.7357	0.6734	0.6973	0.7072	0.6743	0.7252

	SDI&EDI min	all max	all avg	all min
$th = 0.9$	**0.8029**	0.6954	0.7575	0.7917
$th = 0.8$	0.7504	0.6641	0.7279	**0.7531**
$th = 0.7$	0.7488	0.6678	0.7299	**0.7502**
$th = 0.6$	0.7512	0.6695	0.7323	**0.7515**
$th = 0.5$	0.7506	0.6718	0.7344	**0.7515**

The bold values are the highest scores in each column.

Table S11 The PPV scores of integrated evaluation metrics in the archaea data set.

	EMI&SDI max	EMI&SDI avg	EMI&SDI min	EMI&EDI max	EMI&EDI avg	EMI&EDI min	SDI&EDI max	SDI&EDI avg
$M = 100$	0.980	0.980	0.960	**0.990**	**0.990**	0.960	0.980	0.970
$M = 500$	0.788	0.790	0.714	0.766	0.776	0.656	0.768	0.778
$M = 1000$	0.576	0.583	0.489	0.517	0.530	0.445	0.514	0.527
$M = 5000$	0.180	0.186	0.164	0.174	0.180	0.147	0.180	0.183
$M = 10000$	0.108	0.114	0.102	0.109	0.112	0.092	0.112	0.115

	SDI&EDI min	all max	all avg	all min
$M = 100$	0.970	**0.990**	**0.990**	0.970
$M = 500$	0.748	0.804	**0.822**	0.738
$M = 1000$	0.491	0.569	**0.589**	0.503
$M = 5000$	0.176	0.193	**0.203**	0.173
$M = 10000$	0.118	0.116	**0.125**	0.109

The bold values are the highest scores in each column.

Table S12 The PPV scores of integrated evaluation metrics in the micrococcales data set.

	EMI&SDI max	EMI&SDI avg	EMI&SDI min	EMI&EDI max	EMI&EDI avg	EMI&EDI min	SDI&EDI max	SDI&EDI avg
$M = 100$	0.950	0.950	0.950	0.950	0.960	**0.970**	0.950	0.950
$M = 500$	0.850	0.844	**0.854**	0.790	0.806	0.820	0.808	0.800
$M = 1000$	0.687	**0.701**	0.682	0.637	0.645	0.656	0.661	0.668
$M = 5000$	0.213	0.221	**0.224**	0.204	0.208	0.222	0.196	0.200
$M = 10000$	0.125	0.131	0.136	0.121	0.124	**0.137**	0.115	0.117

	SDI&EDI min	all max	all avg	all min
$M = 100$	0.950	0.950	0.950	0.950
$M = 500$	0.732	0.840	0.848	0.828
$M = 1000$	0.516	0.685	0.696	0.643
$M = 5000$	0.194	0.210	0.215	0.217
$M = 10000$	0.117	0.124	0.129	**0.137**

The bold values are the highest scores in each column.

Table S13 The PPV scores of integrated evaluation metrics in the fungi data set.

	EMI&SDI max	EMI&SDI avg	EMI&SDI min	EMI&EDI max	EMI&EDI avg	EMI&EDI min	SDI&EDI max	SDI&EDI avg
$M = 100$	0.120	0.250	0.190	0.190	0.220	0.230	0.120	0.160
$M = 500$	0.056	**0.126**	0.120	0.070	0.088	0.092	0.042	0.090
$M = 1000$	0.035	**0.085**	0.076	0.042	0.067	0.063	0.029	0.060
$M = 5000$	0.016	**0.035**	0.034	0.018	0.023	0.023	0.019	0.026
$M = 10000$	0.013	**0.024**	0.023	0.014	0.016	0.016	0.016	0.016

	SDI&EDI min	all max	all avg	all min
$M = 100$	0.130	0.150	**0.290**	**0.290**
$M = 500$	0.082	0.060	0.120	0.106
$M = 1000$	0.062	0.038	0.081	0.074
$M = 5000$	0.024	0.019	0.031	0.029
$M = 10000$	0.018	0.015	0.021	0.019

The bold values are the highest scores in each column.

Table S14 The AUPR scores of integrated evaluation metrics in the archaea data set.

	EMI&SDI max	EMI&SDI avg	EMI&SDI min	EMI&EDI max	EMI&EDI avg	EMI&EDI min	SDI&EDI max	SDI&EDI avg
$th = 0.9$	0.116	0.120	0.103	0.121	0.124	0.092	0.127	0.131
$th = 0.8$	0.074	0.077	0.066	0.073	0.075	0.059	0.074	0.077
$th = 0.7$	0.053	0.055	0.046	0.052	0.054	0.043	0.053	0.056
$th = 0.6$	0.043	0.045	0.038	0.044	0.045	0.037	0.046	0.049
$th = 0.5$	0.042	0.044	0.037	0.045	0.046	0.039	0.048	0.051

	SDI&EDI	all	all	all
	min	max	avg	min
$th = 0.9$	0.127	0.129	**0.136**	0.111
$th = 0.8$	0.075	0.079	**0.085**	0.071
$th = 0.7$	0.055	0.057	**0.062**	0.051
$th = 0.6$	0.050	0.048	**0.052**	0.044
$th = 0.5$	**0.053**	0.048	0.052	0.044

The bold values are the highest scores in each column.

Table S15 The AUPR scores of integrated evaluation metrics in the micrococcales data set.

	EMI&SDI	EMI&SDI	EMI&SDI	EMI&EDI	EMI&EDI	EMI&EDI	SDI&EDI	SDI&EDI
	max	avg	min	max	avg	min	max	avg
$th = 0.9$	0.214	**0.226**	**0.226**	0.196	0.207	0.225	0.194	0.196
$th = 0.8$	0.134	**0.141**	0.138	0.123	0.129	0.137	0.121	0.123
$th = 0.7$	0.094	**0.099**	**0.099**	0.089	0.093	0.098	0.087	0.088
$th = 0.6$	0.075	0.079	0.079	0.074	0.077	**0.081**	0.070	0.072
$th = 0.5$	0.066	0.070	0.071	0.070	0.073	**0.076**	0.062	0.064

	SDI&EDI	all	all	all
	min	max	avg	min
$th = 0.9$	0.163	0.212	0.222	0.223
$th = 0.8$	0.103	0.133	0.139	0.137
$th = 0.7$	0.077	0.093	0.098	0.098
$th = 0.6$	0.065	0.075	0.079	0.080
$th = 0.5$	0.062	0.066	0.071	0.074

The bold values are the highest scores in each column.

Table S16 The AUPR scores of integrated evaluation metrics in the fungi data set.

	EMI&SDI	EMI&SDI	EMI&SDI	EMI&EDI	EMI&EDI	EMI&EDI	SDI&EDI	SDI&EDI
	max	avg	min	max	avg	min	max	avg
$th = 0.9$	0.0044	0.0050	0.0024	0.0047	0.0050	0.0021	0.0024	0.0029
$th = 0.8$	0.0041	**0.0046**	0.0025	0.0035	0.037	0.0021	0.0025	0.0029
$th = 0.7$	0.0046	0.0051	0.0030	0.0038	0.0040	0.0024	0.0030	0.0035
$th = 0.6$	0.0052	**0.0057**	0.0037	0.0038	0.0041	0.0029	0.0038	0.0043
$th = 0.5$	0.0062	**0.0068**	0.0051	0.0043	0.0046	0.0038	0.0049	0.0056

	SDI&EDI min	all max	all avg	all min
$th = 0.9$	0.0022	0.0044	**0.0054**	0.0024
$th = 0.8$	0.0025	0.0038	**0.0046**	0.0025
$th = 0.7$	0.0031	0.0044	**0.0053**	0.0031
$th = 0.6$	0.0042	0.0047	0.0056	0.0039
$th = 0.5$	0.0059	0.0055	0.0065	0.0055

The bold values are the highest scores in each column.
